# Supplementary material for: Revealing the benefit of eye motion for acuity under emulated cone loss
Source: eLife. 2026 Oct 29;15:RP111289. doi: 10.7554/eLife.111289 (PMC13719164; doi:10.7554/eLife.111289)
Supplement: Peer review history

# Editor report: eLife Assessment

**DOI:** 10.7554/eLife.111289.3.sa0

**Authors:** Xiaorong Liu

**Affiliations:** University of Virginia United States

**Keywords:** Compelling, Important

This short report is an **important** study that visual acuity declines nonlinearly with cone dropout, while eye motion partially compensates by improving sampling from remaining cones. The method for experimentally simulating cone dropout is **compelling**, leveraging state-of-the-art imaging and testing in human subjects.

# Referee report: Reviewer #1 (Public review)

**DOI:** 10.7554/eLife.111289.3.sa1

The authors demonstrate an innovative approach to investigate the effect of cone dropout on visual acuity using their newly developed Oz platform. By systematically reducing the coverage of real-world input to the cone photoreceptor mosaic ("cone dropout condition"), the authors are able to assess how having less cones leads to reduced vision, in comparison to existing approaches ("pixel dropout condition").

The observation of visual acuity maintenance with cone dropout has been a longstanding mystery since the 2013/2018 papers by Ratnam and Foote. The authors should be commended for their approach to address this important question. However, there are some simplifications and assumptions being applied to make this jump (i.e. that a 50% reduction in cone stimulation in a healthy eye is comparable to a 50% reduction in cone density in a patient). It seems unlikely that in a patient eye, with cone dropout, that there will be gaps in the mosaic. Not considering any other non-photoreceptor related reasons for visual acuity loss which can occur in patients, the cone aperture acceptance angle may be different due to changes in cone size or packing; the sensitivity of individual cones may also be reduced due to deficits in the visual cycle recovery which could be affected in disease. Some of these limitations could be addressed and acknowledged more explicitly.

The capture of a rich dataset including both cone imaging and eye motion is valuable. Since the C stimulus test relies on foveal fixation, and there is a high degree of subject-to-subject variation in peak cone density, the authors may wish to report on peak cone density measurements of the subjects being included in this study. In addition, evaluating whether the eye motion is affected by simulated cone dropout condition can help to rule out whether these observed effects can be attributed to eye motion.

Overall, this is an impressive study incorporating state-of-the-art technology to probe the fundamental limits of human vision.

Comments on revised version.

The authors have nicely addressed my concerns. The additional clarifications and revised text have strengthened the paper. Thank you also for pointing out the inaccuracy of referring to the system as the olo system; this has been corrected.

# Author comment: Author response

**DOI:** 10.7554/eLife.111289.3.sa2

**Authors:** Hannah K Doyle, James Fong, Ren Ng, Austin Roorda

**Affiliations:** University of California, Berkeley Berkeley United States; University of California, Berkeley Berkeley United States; University of California, Berkeley Berkeley United States; University of California, Berkeley Berkeley United States

The following is the authors’ response to the original reviews.

**Public Reviews:**

**Reviewer #1 (Public review):**

The authors demonstrate an innovative approach to investigate the effect of cone dropout on visual acuity using their newly developed olo system. By systematically reducing the coverage of real-world input to the cone photoreceptor mosaic ("cone dropout condition"), the authors are able to assess how having fewer cones leads to reduced vision, in comparison to existing approaches ("pixel dropout condition").

The capture of a rich dataset, including cone imaging and eye motion, is valuable. Benchmarking with the prior literature, suggesting that good visual acuity can be maintained despite a 50% loss in cone density, is impressive. However, it is known that cone density varies dramatically from the peak cone density location in the foveal center to even a location a few degrees outside of the fovea. In addition, there is a high degree of subject-to-subject variation in peak cone density. Given that the C stimulus is hollow in the middle, the stimulus does not actually hit the location of the peak cone density but must land slightly outside of it. Therefore, considering the actual cone density of where the stimulus lands will be important to discuss and/or analyze.

The reviewer is correct that the cone density will vary dramatically with distance from the foveal center. However, importantly, in our experiment the Landolt C stimulus is fixed in the world and the eye is free to move across it. Therefore, the subject can direct their gaze to any part of the letter, rather than it being fixed to the hollow center of the letter. In the worst case, if the subject kept their gaze fixed at the center of the letter and were viewing the largest letter corresponding to the worst acuity measured in our experiments (20/100), the cone density on average would be 13% lower at the edge of the letter than at the center. However, it is unlikely that a subject would have fixated in this manner, and the vast majority of letters shown during the experiments were much smaller than 20/100.

For completeness, we have calculated the peak cone densities for each of our subjects using the cone density centroid method described by Reiniger et al (2021). We have added these numbers and a description of the method to the Subjects section in Methods and Materials on lines 339-344.

The observation of visual acuity maintenance with cone dropout has been a longstanding mystery since the 2013/2018 papers by Ratnam and Foote. The authors should be commended for their approach to addressing this important question. However, there are some simplifications and assumptions being applied to make this jump (i.e., that a 50% reduction in cone stimulation in a healthy eye is comparable to a 50% reduction in cone density in a patient). It seems unlikely that, in a patient's eye, with cone dropout, there will be gaps in the mosaic. Not considering any other non-photoreceptor-related reasons for visual acuity loss, which can occur in patients, the cone aperture acceptance angle may be different due to changes in cone size or packing; the sensitivity of individual cones may also be reduced due to deficits in the visual cycle recovery, which could be affected in disease. Some of these limitations could be addressed and acknowledged more explicitly.

Cone loss does manifest differently in different retinal degenerative diseases, and in this work we implement dropout on a cone-by-cone level. To address the reviewer’s points, we have added a description of the range of spatial manifestations of cone loss across a range of diseases to the Discussion section on lines 320-328, and emphasize that we focus on one particular manifestation in this paper.

Overall, this is an impressive study incorporating state-of-the-art technology to probe the fundamental limits of human vision.

We thank the reviewer for their helpful comments and constructive feedback.

**Recommendations for the authors:**

**Reviewer #1 (Recommendations for the authors):**

The patient recruitment limitation seems to be a bit artificial here. This is indeed a limitation, but perhaps not the primary limitation or motivating factor. Consider removing/rephrasing this motivation.

We agree with the reviewer’s comment, and have removed it from the abstract and removed its framing as a limitation in the Introduction section in two instances on lines 31 and 36-37.

Was the peak cone density quantified, and the location of the peak cone density determined? Reporting the range of eccentricities over which the C stimulus lands relative to the peak cone density location, as well as the actual cone density that is being used to sample the C stimulus on the retina, seems to be important for contextualizing this study. It is a bit too simple to only consider the percentage of cones that are reduced.

We have added the peak cone density for each subject to the Subjects section in Methods and Materials. This experiment did not require fixation; rather, the Landolt C stimulus was fixed in space and the subject could move their eye freely across it, meaning that different parts of the fovea may have sampled the letter on different trials. At the highest dropout percentage, where acuity was the worst, the letter size was 20/100 at threshold, or 25 arcmin. If the subject were to fixate with their peak cone density at the center of the letter, we have computed that the average decrease in cone density at the edge of the letter (12.5 arcmin away) would be 13%. The majority of trials in the experiment showed letters that were much smaller than this, and would have been subject to even less variation in cone density.

Do the authors have any idea about the approximate size of the cones in healthy subjects compared to diseased eyes? Importantly, if the cones in patients are larger due to the dropout of their neighbors, then the retinal coverage area would be larger due to their larger size, and the amount of light that can be coupled into larger cones may also be larger. Can this be modeled or discussed?

In our implementation, we did not emulate a change in cone size, and rather modeled the loss as discrete holes in an otherwise intact retina. We have added text to the Discussion section on lines 320-328 to make the distinction between this form of cone loss and other forms where cones appear to fill in for their neighbors resulting in a contiguous mosaic of lower density overall.

Acknowledging some of the shortcomings of this approach for simulating the patient condition could be improved. It may be worthwhile to tone down the premise of this paper if these cannot be adequately explained.

In order to tone down the premise of the paper, we have made the following changes to the text.

We now emphasize on lines 65-69 in the Introduction section that we focus specifically on the impact of cone loss on acuity without modeling downstream factors.

In addition to the description of other diseases that we added in response to a previous comment, we have also added the following text on lines 313-318 of the Discussion section:

“... factors beyond the photoreceptors also play a role in shaping vision under retinal degeneration. In this work, we did not model any downstream factors such as shorter outer segments (Foote 2018), retinal rewiring (Jones 2016, Lee 2021), or ganglion cell hyperactivity (Kramer 2023). Instead, we sought to characterize vision in the presence of cone loss at the lowest possible level, considering only the decrease in sampling power at the retinal input.”

In the methods, it is not completely clear the rationale for determining the appropriate size of the C stimulus. How is visual acuity determined if the C stimulus size is not changed?

A more careful explanation of how the C stimulus size is set is warranted.

In the experiments measuring visual acuity, the C stimulus size was selected by a QUEST staircase on each trial. For each dropout condition, we ran 4 interleaved QUEST staircases with 20 trials each. This is described in the “Acuity Threshold Experiment” section in the main text (lines 99-100) and in Materials and Methods (line 407). To clarify further, we have updated the following sentence on line 423:

“For each condition, we ran 4 interleaved QUEST staircase procedures (Watson and Pelli, 1983) with 20 trials per staircase, which varied the size of the Landolt C on each trial.”

What is the clinical visual acuity of the subjects being tested? It seems important to report this if the authors want to use their C stimulus as a proxy for clinical visual acuity.

The subjects being tested have excellent acuity. In Figure 1, we can see that their adaptive-optics-corrected acuity for the baseline 0% dropout condition ranges from approximately 20/10 to 20/12.5 across the 4 subjects. We have added the following statement to the “Subjects” section in Materials and Methods (line 339):

“All subjects self-reported to have normal vision.”

Given that the title of the paper emphasizes the role of eye motion, it seems that a more careful analysis of the magnitude and type(s) of eye motion could be added. There are eye motion data provided in the supplemental figure, but it is not completely clear how this eye motion data is actually being used to derive meaningful information about visual acuity.

We performed analyses to determine whether there seemed to be a significant difference in eye motion patterns between the cone and pixel dropout conditions, which was described in the section “Analysis of Eye Motion Data” and in Supplementary Figure S1. In that figure, we show that for all 4 subjects there is no significant difference in the iso-density contour area containing 68% of their eye motion data. We suggest in the paper that due to the pseudorandom presentation of trials and the limited duration of those trials, subjects were unlikely to adapt or adjust their eye movement, and that instead their natural eye motion served as a data collector that improved acuity.

What is the accuracy of the eye motion and cone dropout stimulation delivery in the fovea? Given the small size of the cones, it seems that this is one of the most challenging locations of the eye to test with this new olo technology.

Eye tracking and targeted light delivery are crucial in the AOSLO system and the reviewer is correct to point out that this is most difficult to achieve at the foveal center. To address this concern, we have done some simple modeling and have added the following text to the Cone-by-Cone Stimulation section in the Methods and Materials.

“This latency, combined with other factors such as diffraction and residual aberrations, limit the ability to restrict the light to only the targeted cone. Considering a 543-nm focus through a 7.2 mm pupil, a random tracking error with a full-width-at-half maximum (FWHM) of 0.5 arcminutes (Harmening et al. (2014)), a 0.0125 diopter residual defocus error (maximum error given the step sizes of 0.025 diopters in the AOSLO defocus controller), an average cone spacing of 0.5 arcminutes (Wang et al. (2019)), and a Gaussian cone acceptance aperture with a FWHM that is 0.5 times the inner segment diameter (Macleod et al. (1992)), we estimate that each targeted cone receives 5.41 times more light than its nearest neighbor. This means that the ’dead’ cones cannot be fully excluded from the visual processing. Furthermore, the light leakage reported in Fong et al. (2025) further adds to the signal of non-targeted cones.

Nevertheless, it is important to point out that the information about the stimulus (Landolt C in our case) is sampled at the targeted cone’s location and so, although nearby stimulated cones might detect light, they do not contribute to any increases in the sampling process. This is analogous to adding defocus blur to letters in the pixel dropout condition as neither situation will improve the spatial information.”

References

Reiniger, J.L., Domdei, N., Holz, F.G., Harmening, W.M.: Human gaze is systematically offset from the center of cone topography. Current Biology 31(18), 4188–4193 (2021)
